# Effect of Surfactant Therapy on Clinical Outcomes of COVID-19 Patients With ARDS: A Systematic Review and Meta-Analysis

**DOI:** 10.7759/cureus.56238

**Published:** 2024-03-15

**Authors:** Maneeth Mylavarapu, Venkata Vamshi Krishna Dondapati, Sriharsha Dadana, Dhruvikumari D Sharma, Bhaswanth Bollu

**Affiliations:** 1 Preventive Medicine, Adelphi University, Garden City, USA; 2 Medicine and Surgery, Institute of Medical Sciences, Varanasi, IND; 3 Internal Medicine, Cheyenne Regional Medical Center, Cheyenne, USA; 4 Biochemistry, Spartan Health Sciences University, Vieux Fort, LCA; 5 Medicine, Avalon University School of Medicine, Willemstad, CUW; 6 Emergency Medicine, All India Institute of Medical Sciences, New Delhi, IND

**Keywords:** meta-analysis, length of hospitalization, mortality, ards, covid-19, surfactant therapy

## Abstract

Introduction: The COVID-19 pandemic has brought unprecedented challenges, not only in terms of public health but also in the realm of innovative therapeutic approaches to combat the severe respiratory complications associated with the virus. The effect of surfactant therapy on reducing mortality in COVID-19 patients with acute respiratory distress syndrome (ARDS) hasn't been explored before.

Methods: We conducted a search on PubMed, Scopus, Science Direct, and Clinicaltrials.gov to identify relevant studies, incorporating subject headings and keywords related to "Surfactant Therapy," "COVID-19," and "ARDS." Binary random effects were used to estimate the odds ratio (OR) for 28-day mortality, and continuous random effects were used to estimate the mean difference (MD) for length of hospitalization with their respective 95% confidence interval (CI). Analysis was performed with RevMan Version 5.4.1 (The Cochrane Collaboration, London, GBR).

Results: We included four studies with 126 patients. Patients who received surfactant had lower odds of mortality (OR 0.53, 95% CI (0.23, 1.20), p=0.13) and a shorter duration of hospital stay (MD -5.69, 95% CI [-7.06, -4.30], p <0.00001) compared to patients who did not receive surfactant therapy. However, the findings regarding mortality were not statistically significant.

Conclusions: The COVID-19 patients with ARDS who received surfactant therapy had lower hospitalization stays and mortality rates, indicating that surfactant therapy may improve clinical outcomes in COVID-19 patients with ARDS. However, the results were not significant, and further research with more prospective studies and randomized clinical trials (RCTs) with larger sample sizes is needed to confirm these findings and assess their practical significance and generalizability.

## Introduction and background

In the wake of 2019, a novel coronavirus emerged in Wuhan, China, rapidly causing severe respiratory syndrome and fatal pneumonia. Within three months, the WHO officially designated the outbreak as a global pandemic induced by SARS-CoV-2, leading to the disease now known as COVID-19 [1]. This pandemic plunged societies into disarray, challenging healthcare systems and pushing the boundaries of scientific understanding, economic advancements, and social unity on a global scale [2-4]. One of the biggest RNA genomes ever discovered is that of the family *Coronavirinae*, which consists of enveloped positive single-stranded RNA [5,6]. Alphacoronavirus, betacoronavirus, gammacoronavirus, and deltacoronavirus are the four genera into which they may be subdivided. Of these, betacoronavirus (SARS-CoV-2 genera) is the most pathogenic subtype for humans [7-9]. Patients with the infection suffer from flu-like symptoms, including exhaustion, fever, coughing, and nasal congestion [10,11]. As the infection worsens, patients develop dyspnea and other viral pneumonitis symptoms, such as reduced oxygen saturation and lymphopenia, along with intralobular involvement in ground-glass opacities and alveolar exudates on chest imaging. These patients' imaging resembles that of severe acute lung damage conditions such as acute respiratory distress syndrome (ARDS) [12].

The symptoms of ARDS are respiratory distress related to hypoxemia and bilateral infiltrates on chest imaging. Acute respiratory distress syndrome was first described by Ashbaugh et al. [13] in 1967. Since then, there has been discussion over the definition of the diagnosis and its criteria because there isn't a gold standard. The Berlin definition established criteria that are briefly highlighted in ARDS: 1) sudden start of respiratory symptoms; 2) bilateral infiltration on chest imaging, when cardiac disease or fluid overload cannot entirely account for pulmonary edema; and 3) hypoxemia, which is graded into three groups [14]. The pathophysiological features of acute and diffuse inflammatory damage to the alveolar-capillary barrier are linked to increased vascular permeability, decreased compliance, and a smaller amount of aerated lung tissue, all compromising gas exchange and resulting in hypoxemia [15]. Studies have demonstrated increased secretory phospholipase A2 (sPLA2) levels in the bronchoalveolar lavage fluid of ARDS patients, which degrades phospholipids (components of lung surfactant) [16,17]. However, replacing surfactant, i.e., exogenous surfactant therapy, wasn't considered an effective adjuvant therapy for adult ARDS as no significant improvement was seen in mortality [18]. Nevertheless, no meta-analysis was done regarding the effect of surfactant therapy on clinical outcomes in COVID-19 ARDS. This systematic review and meta-analysis aim to shed light on the impact of surfactant therapy on the clinical outcomes of ARDS in COVID-19 patients.

## Review

Methods

**Figure 1:**
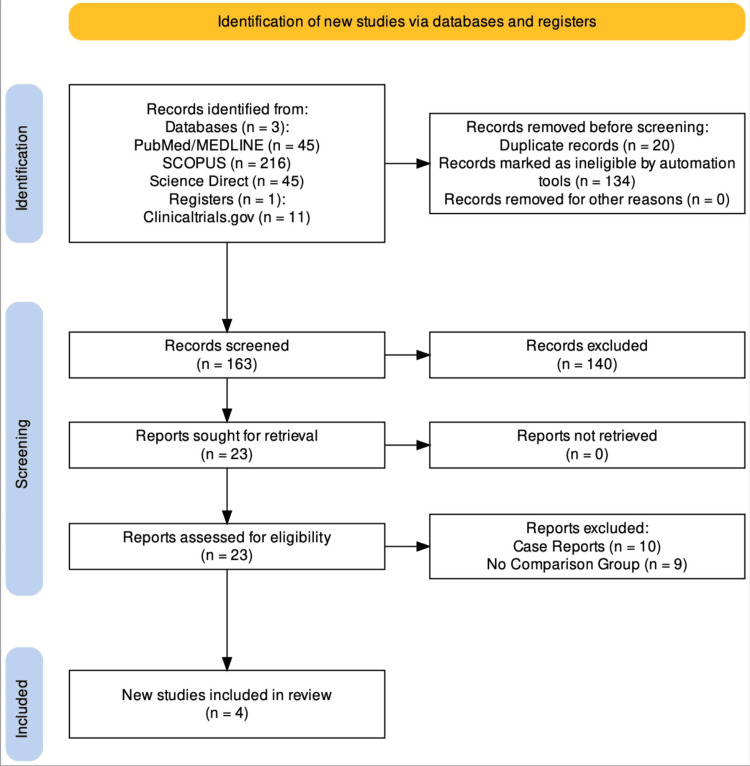
PRISMA flow chart of the study selection process PRISMA: Preferred Reporting Items for Systematic Reviews and Meta-analyses

The study was carried out following the Preferred Reporting Items for Systematic Reviews and Meta-analyses (PRISMA) guidelines [19]. A comprehensive literature search was conducted in several prominent reliable databases, including PubMed/MEDLINE, Scopus, clinicaltrails.gov, and Science Direct, for relevant literature. Subject headings and keywords related to "Pulmonary surfactants," "Surfactant Therapy," "ARDS," and "COVID-19" were included. The search terms were effectively combined using suitable Boolean operators. The references to the selected studies were also examined to verify the comprehensiveness of the search. The search strategy utilized for the study is outlined in Table 1. All the studies comparing the clinical outcomes of patients with COVID-19 ARDS with and without surfactant therapy were included. A detailed list of inclusion and exclusion criteria is outlined in Table 2. The study protocol was registered in the International Prospective Register of Systematic Reviews (PROSPERO database; ID no.: CRD42023448696). The process of screening the title, abstract, and full text was done independently by two reviewers, VK and BB. Conflicts concerning the screening were resolved by the third reviewer, MM. Figure 1 depicts the PRISMA flow chart outlining the study selection process [20].

**Table 1:** Search strategy

PubMed:
("Surfactant therapy" OR "Pulmonary surfactants" OR "Exogenous surfactant" OR "Surfactant replacement therapy") AND ("COVID-19" OR "Coronavirus Disease 2019" OR "SARS-CoV-2" OR "Severe acute respiratory syndrome coronavirus 2") AND ("ARDS" OR "Acute lung injury" OR "Respiratory distress syndrome, adult") AND ("Clinical outcomes" OR "Treatment outcomes" OR "Patient outcomes" OR "Therapeutic effects")
Science Direct:
("Surfactant therapy") AND ("COVID-19") AND ("ARDS" OR "Respiratory distress syndrome, adult") AND ("Clinical outcomes" OR "Treatment outcomes" OR "Patient outcomes" OR "Therapeutic effects")
SCOPUS:
("Surfactant therapy") AND ("COVID-19") AND ("ARDS" OR "Respiratory distress syndrome, adult")
Clinicaltrials.gov (search terms)
Surfactant Therapy; COVID-19; ARDS; Clinical Outcomes

**Table 2:** Inclusion and exclusion criteria SR: Systematic review, MA: Meta-analyses, NRS: Non-randomized studies

Inclusion criteria	Exclusion criteria
1. Articles containing data about surfactant therapy and clinical outcomes of patients with COVID-19	1. No data on either treatment by surfactant or clinical outcomes
2. English language literature	2. Non-English literature
3. Age ≥ 18 years	3. Grey literature (book chapters, dissertations, etc.) and secondary studies (SRs, MAs, and NRS)
4. Prospective or retrospective studies (with control group)	4. Studies without a control group

**Figure 2:**
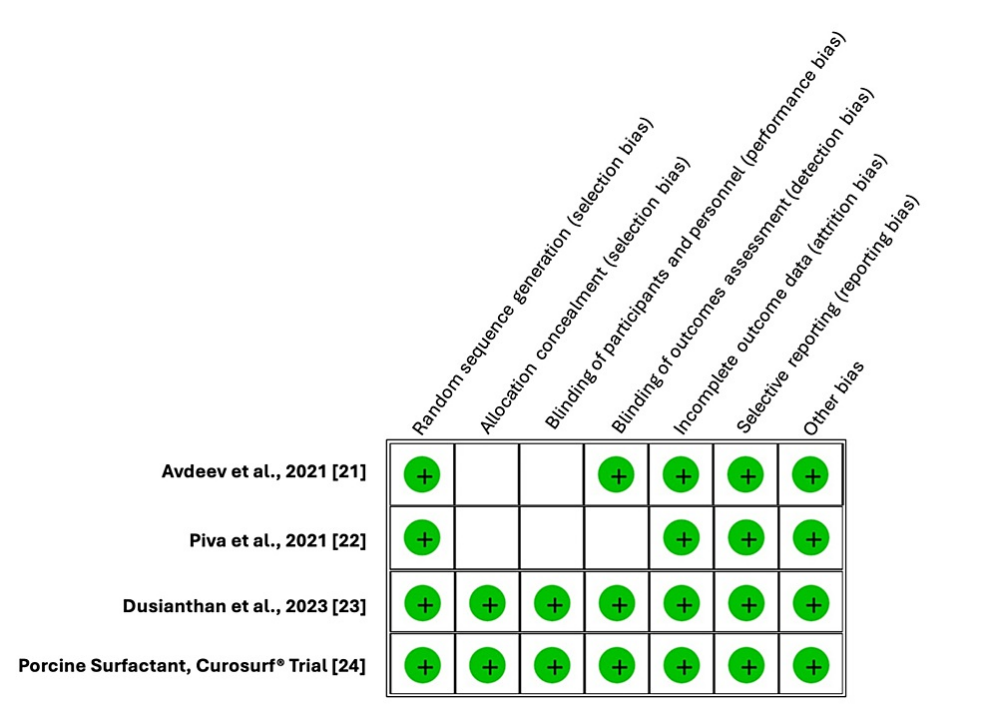
Risk of bias assessment of included studies The green colored circle indicates a low risk of bias, and the blank boxes indicate unclear risk.

Regarding the quality appraisal of the studies, each of the included studies’ methodological quality was evaluated separately by two reviewers, SD and DD. Furthermore, the risk of bias assessment in the included studies was assessed, and statistical analysis was done using the critical appraisal tools of RevMan version 5.4.1 (The Cochrane Collaboration, London, GBR) (Figure 2). Binary random effects were used to estimate the odds ratio (OR) for 28-day mortality, and continuous random effects were used to estimate the mean difference (MD) for length of hospitalization with their respective 95% confidence interval (CI). The I² statistic was used to assess for heterogeneity. Funnel plots were used to assess publication bias.

Results

**Figure 3:**
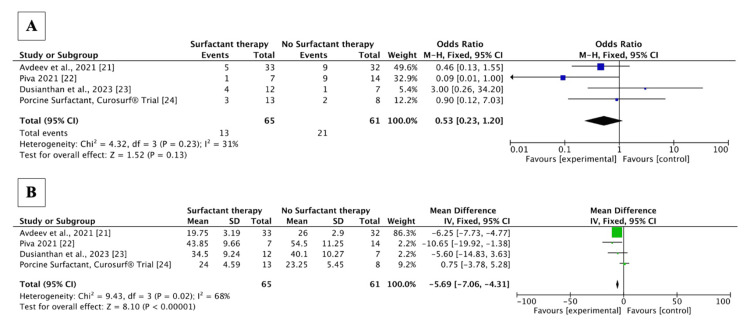
Forest plots showing the effect of surfactant therapy A: Forest plots explaining the effect of surfactant therapy on mortality in patients with COVID-19 ARDS; B: Forest plots explaining the effect of surfactant therapy on length of hospitalization in patients with COVID-19 ARDS ARDS: Acute respiratory distress syndrome, IV: Inverse variance, M-H: Mantel–Haenszel

**Figure 4:**
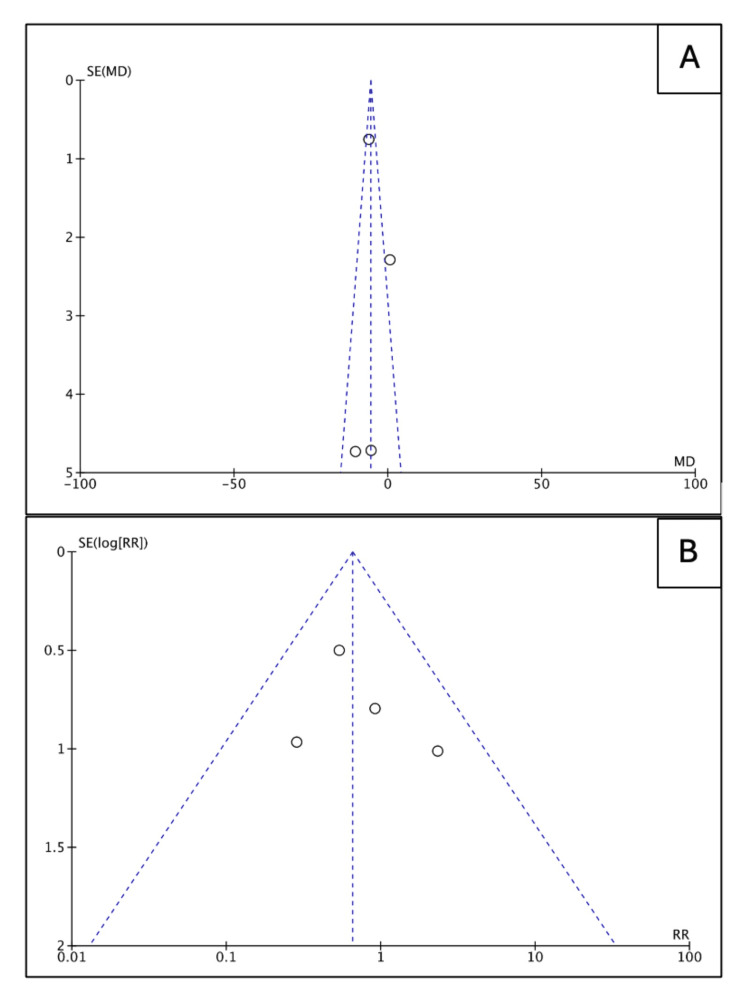
Publication bias assessment A: Funnel plot depicting no significant publication bias regarding mortality in the studies included [21-24]; B: Funnel plot depicting no significant publication bias concerning the length of hospitalization in the studies included [21-24] SE: Standard error, MD: Mean difference, RR: Relative risk

We included four studies [21-24] with 126 patients; 41 (32.53%) patients were females, and 65 (51.6%) patients were treated with surfactant therapy. Table 3 outlines the baseline characteristics of the included studies. Patients who received surfactant had lower odds of 28-day mortality (OR 0.53, 95% CI (0.23, 1.20), p=0.13) and shorter duration of hospital stay (MD -5.69, 95% CI (-7.06, -4.30), p <0.00001) compared to patients who did not receive surfactant therapy (Figure 3). However, the findings regarding mortality were not statistically significant. Funnel plots (Figure 4) indicate that the publication bias was insignificant.

**Table 3:** Baseline characteristics of the included studies ST: Surfactant therapy; C: Control group; SD: Standard deviation

Author	Type of study	Total sample (n)	Cohort	Age (years)		Females	Mortality
				Mean age	SD		
Avdeev et al., 2021 [20]	Prospective	65	ST (33)	60	4.6	15	5
			C (32)	61.5	5.2	9	9
Piva et al., 2021 [21]	Retrospective	21	ST (7)	66.1	4.33	1	1
			C (14)	60.85	10.79	3	9
Dusianthan et al., 2023 [22]	Prospective	19	ST (12)	55.5	14	5	4
			C (7)	56.5	11.2	3	1
Porcine Surfactant, Curosurf® Trial, 2023 [23]	Prospective	21	ST(13)	63.6	9.2	3	3
			C (8)	51	18.2	2	2

Discussion

Our study findings suggest a potential benefit associated with surfactant therapy. Specifically, in patients who were treated with surfactant therapy, we found a decrease in mortality and duration of hospitalization. The possibility of these results could be explained by the pathophysiological mechanisms by which SARS-CoV-2 infection causes ARDS. Acute respiratory distress syndrome can develop in critically ill COVID-19 patients with deteriorating respiratory function. The Berlin criteria shed light on a peculiar difference between the SARS-CoV-2-induced ARDS and the 'normal' ARDS. An increased host inflammatory response appears to be the root cause of acute lung impairment in SARS-CoV-2 infections. The cytokine storm and intravascular coagulation could both contribute to the development of microthrombi in the pulmonary arteries, which ultimately disrupts the self-healing capacities of the lungs, precipitating the ARDS and predisposing patients to fibrosis. However, the inflammatory concept cannot fully explain the pathophysiology of COVID-19-induced lung injury [16,17].

The SARS-Cov-2 infection of the alveolar type 2 (AT2) cells of the host, facilitated by the angiotensin-converting enzyme 2 (ACE2) gene, serves as the catalyst and leads to a cascade of phenomena that ultimately lead to progressive lung injury. This leads to subsequent thrombosis in the capillary bed, causing atelectasis and a break in the alveolar-capillary barrier. This ongoing AT2 degradation inhibits the production of surfactants. Furthermore, the SARS-Cov-2 virus also targets type 2 pneumocytes, causing further decline in surfactant production and further precipitating ARDS [16,17,25]. Additionally, studies state that COVID-19 ARDS can have a similar presentation to ARDS in premature infants. Initially, patients experience low oxygen levels alongside sustained lung compliance, called the L-type presentation. However, these patients often transition to the second presentation, called the H-type. Such a shift only occurs due to significant damage to the AT2 cells and the depletion of phosphatidylcholine. The H-type presentation results in high stiffness (reduced compliance), resembling a premature infant's lungs (lack of sufficient surfactant production) [25-27].

Recently, several case reports, case series, and observational studies have reported the use of exogenous surfactants in COVID-19 patients with ARDS, which improved clinical outcomes [28-30]. However, our study serves as the first meta-analysis to examine the effects of surfactant therapy on the clinical outcomes of ARDS in COVID-19 patients. The limitations of the study include a low sample population and non-significant results on mortality. The potential reasons could be heterogeneity in the patient population, variations in the timing of the surfactant therapy (early or late), delivery methods (nebulized, intratracheal, or intrabronchial), varied severity of illness and protocol of the standard of care followed before the surfactant therapy, and ideal time intervals between the doses. Additionally, studies have reported that multiple clinical trials of surfactant therapy for adult ARDS failed due to the rapid turnover of exogenous surfactants [31]. The effect of rapid turnover could also contribute to the non-significant results observed in our study. Future clinical trials with large cohorts need to be conducted to further evaluate the effect of surfactant therapy on clinical outcomes in COVID-19 ARDS and also to find the ideal dose, route of delivery, and the time interval between multiple doses. Furthermore, studies also need to evaluate the safety and long-term effects of surfactant therapy in COVID-19 ARDS patients.

## Conclusions

Our meta-analysis suggests a potential benefit with surfactant therapy for COVID-19 patients with ARDS. However, the results did not demonstrate statistical significance for mortality. Therefore, further research needs to be conducted to evaluate the safety and efficacy of surfactant therapy in patients with COVID-19 ARDS.
